# A phase I clinical trial of binimetinib in combination with FOLFOX in patients with advanced metastatic colorectal cancer who failed prior standard therapy

**DOI:** 10.18632/oncotarget.19336

**Published:** 2017-07-18

**Authors:** May Cho, Jun Gong, Paul Frankel, Timothy W. Synold, Dean Lim, Vincent Chung, Joseph Chao, Daneng Li, Yuan Chen, Stephen Sentovich, Kurt Melstrom, Gagandeep Singh, Eloise Luevanos, Marwan Fakih

**Affiliations:** ^1^ Department of Medical Oncology, City of Hope National Medical Center, Duarte, CA, USA; ^2^ Department of Statistics, City of Hope National Medical Center, Duarte, CA, USA; ^3^ Department of Cancer Biology, Beckman Research Institute of City of Hope, Duarte, CA, USA; ^4^ Department of Molecular Medicine, Beckman Research Institute of City of Hope, Duarte,CA, USA; ^5^ Department of Surgical Oncology, City of Hope National Medical Center, Duarte, CA, USA

**Keywords:** binimetinib, MEK inhibitor, FOLFOX, metastatic colorectal cancer, refractory disease

## Abstract

**Background:**

This was a first in-human, open-label, dose-escalation phase I study conducted to evaluate the maximum tolerated dose (MTD), safety, and efficacy of the combination of oral binimetinib and FOLFOX.

**Materials and Methods:**

Patients with metastatic colorectal cancer (mCRC) who progressed on prior standard therapies received twice daily binimetinib continuously or intermittently with FOLFOX. Dose-limiting toxicities (DLTs) were assessed in the first 2 cycles of study treatment. Pharmacokinetic (PK) analysis of 5-FU and oxaliplatin was performed at the MTD in an expanded 6 patient cohort.

**Results:**

Twenty-six patients were enrolled and assessed for safety. In the dose-escalation phase, no DLTs were noted in all binimetinib dosing schedules and the MTD of binimetinib in with FOLFOX was 45 mg orally twice daily. There were no significant differences in the PKs of 5-FU or oxaliplatin with or without binimetinib. Continuous dosing of binimetinib produced SD at 2 months in 9 of 13 evaluable patients and a median PFS of 3.5 months. Nine of 10 patients had PD at 2 months on the intermittent arm.

**Conclusions:**

Oral binimetinib and FOLFOX has a manageable toxicity profile and showed some evidence of antitumor activity in heavily pretreated mCRC patients.

## INTRODUCTION

Colorectal cancer (CRC) remains the third leading cause of cancer death in both men and women in the U.S. with an estimated 49,190 dea1ths to occur in 2016 [1]. The integration of combination cytotoxic therapy and targeted agents in metastatic CRC (mCRC) has improved median overall survival (OS) to nearly 30 months from approximately 12 months in the era of fluoropyrimidine monotherapy [2]. Despite this, it appears that a plateau in OS has been reached in mCRC treatment that underscores the need for clinical and rational development of novel therapeutics in this population.

Since its approval in 2004, FOLFOX remains a widely used treatment standard in mCRC [3]. Various strategies of intermittent FOLFOX use and FOLFOX rechallenge have been employed to maximize oxaliplatin exposure in mCRC [4–6]. Preclinical evidence has shown that platinum resistance in cisplatin-treated ovarian cancer cells is associated with epithelial mesenchymal transition (EMT), cancer stem cells (CSC)-like changes, and increased activation of extracellular signal-regulated kinase (ERK) 1 and 2 [7, 8]. Pretreatment with a mitogen-activated protein kinase (MAPK)/ERK kinase (MEK) inhibitor decreased cisplatin-induced ERK1/2 activation and resulted in synergistic antitumor activity in ovarian cancer cell lines [7]. MEK inhibition similarly suppressed cisplatin-induced EMT and CSC-like changes, leading to increased cisplatin sensitivity [8]. MAPK and MEK inhibition have been shown to reduce DNA excision repair protein (ERCC1) expression and increase cisplatin sensitivity or reverse cisplatin resistance across several preclinical tumor models [9, 10].

Binimetinib is a novel, potent, and selective ATP-noncompetitive inhibitor of MEK1/2 that has demonstrated antitumor activity across several tumors (including colorectal) *in vitro* and *in vivo* regardless of RAS/RAF pathway mutations [11–13]. Binimetinib inhibits MEK and downstream phosphorylated ERK (pERK) in the nanomolar range and has shown synergistic antitumor activity when combined with cytotoxic chemotherapy on both continuous and intermittent dosing schedules in the preclinical setting [11, 14]. In the first dose-escalation phase I study, the maximum tolerated dose (MTD) of single-agent oral binimetinib was 60 mg twice daily in patients with pretreated advanced solid tumors [15]. Despite a manageable safety profile in other phase I trials in advanced solid tumors, oral binimetinib at 45 mg twice daily became the recommended phase II dose (RP2D) due to recurrent dose-limiting toxicities (DLTs) of retinal events [16, 17]. In a phase II trial of binimetinib 45 mg twice daily, grade 1–2 retinal adverse events (AEs) were seen in 18% of patients with *NRAS*- or *BRAF*-mutated advanced melanomas [18]. Five phase Ib/II trials have established binimetinib 45 mg oral twice daily (continuous dosing) in combination with various targeted agents as the MTD or RP2D [19–24]. In a phase Ib trial of continuous or intermittent binimetinib (days 1–5 weekly for 3 out of 4 weeks) in combination with paclitaxel 80 mg/m^2^ infusion weekly for 3 out of 4 weeks, binimetinib 30 mg orally twice daily and 45 mg orally twice daily became the RP2D for continuous dosing and intermittent dosing, respectively, when combined with paclitaxel [25].

Based on the above rationale, we proposed that MEK inhibition may improve clinical responses and overcome resistance to platinum-based (FOLFOX) therapy in mCRC. We conducted the first in-human, single-arm, open-label phase I clinical trial of binimetinib in combination with FOLFOX in patients with mCRC who have progressed on prior standard therapies. The goal of this study was to determine the feasibility of combining binimetinib and FOLFOX and to identify the MTD of this combination.

## RESULTS

### Study population

From July 16, 2014 to February 11, 2016, a total of 41 patients were screened with a final 26 patients meeting all eligibility criteria and subsequently enrolled in the study. The mean age was 54 years (range 43–78) with the majority of patients being male (69.2%) and of Caucasian (38.5%) or Hispanic (34.6%) race (Table 1). More than half (69.2%) had *KRAS*-mutated colorectal tumors with primary tumors of the right colon comprising 42.3% of the population. The median number of prior therapies was 3 (range 1–5) with the majority (69.2%) having received ≥ 3 prior lines of therapies.

**Table 1:** Patient characteristics

Total patients (*n*)	26
Age (mean)	54 years (range 43–78)
ECOG 0 1	14 (53.8%) 12 (46.2%)
Sex Female Male	8 (30.8%) 18 (69.2%)
Race Asian African American Hispanic White Other	5 (19.2%) 1 (3.8%) 9 (34.6%) 10 (38.5%) 1 (3.8%)
Primary tumor Rectum Left colon Right colon	4 (15.4%) 11 (42.3%) 11 (42.3%)
*RAS* mutation status Wild-type Mutant	8 (30.8%) 18 (69.2%)
Prior lines of therapy 0–1 2 ≥ 3	1 (3.8%) 7 (27.0%) 18 (69.2%)

ECOG, Eastern Cooperative Oncology Group performance status.

### Safety and tolerability

A total of 26 patients were evaluated for toxicities in the study. Sixteen patients were enrolled into the continuous binimetinib dosing arm and 10 patients were enrolled into the intermittent binimetinib dosing arm. A median 6 cycles (range 1–20) of study treatment was administered in the continuous binimetinib dosing + FOLFOX population. A median 4 cycles (range 4–8) of study treatment was administered in the intermittent binimetinib dosing + FOLFOX arm.

Following standard dose-escalation rules, no DLTs were noted in either binimetinib dosing schedules when combined with FOLFOX every 2 weeks in the dose-escalation phase of the study. The MTD of binimetinib in combination with FOLFOX was 45 mg orally twice daily in either continuous or intermittent dosing schedules. No further escalation of binimetinib dosing was allowed by design, given that the single agent recommended dose is 45 mg orally twice daily. Toxicities associated with study treatment are detailed in Table 2. FOLFOX-associated toxicities were consistent with the literature and included bone marrow suppression, neuropathy, gastrointestinal toxicity, and hypersensitivity. In the continuous 30 mg twice daily cohort, 2 patients (67%) had grade 1–2 CPK elevation while 1 (33%) had grade ≥ 3 elevation. Notably, one case of serious ophthalmic toxicity occurred in a 59 year-old male who developed acute loss of vision on his 8th cycle of treatment on 30 mg continuous BID dosing of binimetinib. Ophthalmological examination confirmed a grade 3 retinal vein occlusion (RVO) and vitreous hemorrhage and the patient was taken off study treatment. Complete resolution of symptoms and abnormal retinal findings was confirmed at 3-month follow-up. In the continuous 45 mg oral twice daily arm (including PK cohort), the most common binimetinib-related toxicity was rash (85% grade 1–2, 8% grade 3). Seven patients (54%) had grade 1–2 CPK elevation while 2 (15%) had grade 3 CPK elevation. Three grade 1 ophthalmological toxicities consisted of blurry vision with glaucoma [1], retinopathy with glaucoma (1), and glaucoma (1) occurred during binimetinib dosing on the continuous 45 mg twice daily arm. These did not lead to any dose reductions or interruptions in binimetinib. In the intermittent 45 mg twice daily arm, there were 4 patients (40%) with grade 1–2 CPK elevation, but none with grade ≥ 3 CPK elevation. Three patients (30%) experienced grade 1–2 skin rash, but none had grade ≥ 3 rash. There were no instances of retinal abnormalities in this arm, although 1 patient had grade 1 blurred vision and another had grade 1 cataracts (Table 2).

**Table 2:** Treatment-related (possibly related) adverse events*

Adverse event	Continuous arm 30 mg binimetinib BID + FOLFOX *n* = 3		Continuous arm 45 mg binimetinib BID + FOLFOX *n* = 13**		Intermittent arm 45 mg binimetinib BID + FOLFOX *n* = 10	
	Grade 1–2	Grade 3–4	Grade 1–2	Grade 3–4	Grade 1–2	Grade 3–4
CPK elevation	2 (67%)	1 (33%)	7 (54%)	2 (15%)	4 (40%)	0
LFT abnormality	2 (67%)	0	9 (69%)	0	4 (40%)	0
Rash	2 (67%)	0	11 (85%)	1 (8%)	3 (30%)	0
Anemia	2 (67%)	0	5 (38%)	0	3 (30%)	0
Thrombocytopenia	3 (100%)	0	5 (38%)	1 (8%)	4 (40%)	0
Diarrhea	1 (33%)	0	6 (46%)	0	6 (60%)	0
Fatigue	1 (33%)	0	7 (54%)	0	6 (60%)	0
Neuropathy	1 (33%)	0	7 (54%)	2 (15%)	3 (30%)	0
Nausea/Vomiting	1 (33%)	0	7 (54%)	0	5 (50%)	0
Ocular	0	1 (33%)†	3 (23%)	0	2 (20%)	0
Neutropenia	0	0	1 (8%)	1 (8%)	1 (10%)	1 (10%)
Cardiac Troponin I	0	0	1 (8%)	0	1 (10%)	0
Allergic Reaction	0	0	0	0	2 (20%)	0
Anaphylaxis	0	0	0	1 (8%)	0	0
Ataxia	0	0	0	0	1 (10%)	0

BID, twice daily; FOLFOX, 5-flourouracil, leucovorin, and oxaliplatin; CPK, creatine phosphokinase or creatine kinase; LFT, liver function test.

*One patient on continuous binimetinib 30 mg BID died of respiratory failure from pneumonia considered disease-related; one elderly patient on continuous binimetinib 45 mg BID had a sudden death during cycle 4 of the study and was of undetermined cause.

**One patient on continuous binimetinib 45 mg committed suicide after 4 days. The patient was not evaluable for DLT, but is counted in the denominator for Table 2. †Vitreous hemorrhage. ‡ CD4 lymphocytes low.

### Efficacy

**Figure 1:**
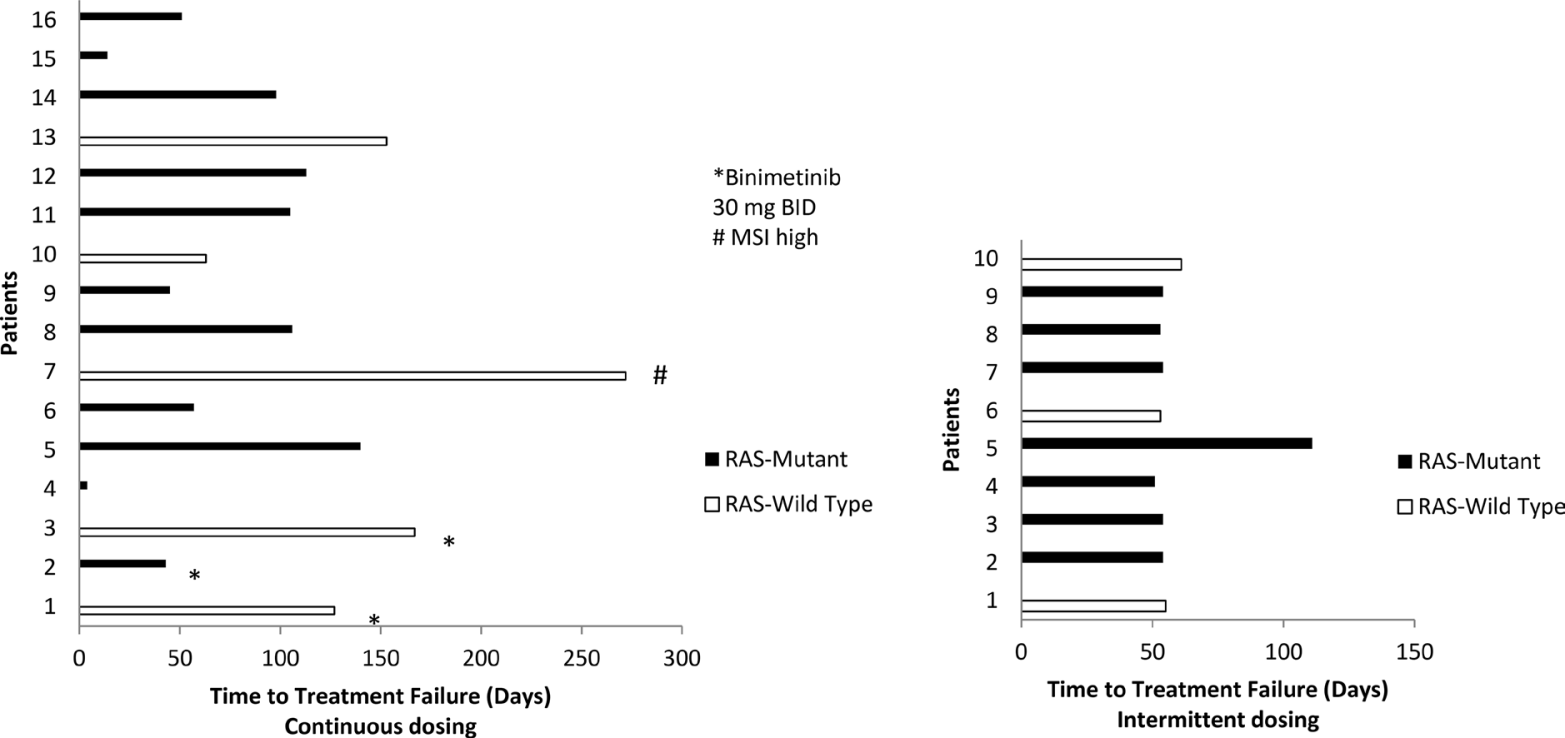
Time to treatment failure by *RAS* mutation status and binimetinib dosing schedule On continuous dosing, reasons to stop treatment included: patient 1 and 15 due to toxicity, patient 4 was a suicide on day 4, patient 5 went to surgery, patient 13 discontinued due to patient’s choice, and the remainder progressed. All patients on the intermittent arm stopped for PD.

A total of 23 patients, 3 enrolled in the 30 mg binimetinib continuous arm, 10 in the 45 mg binimetinib continuous arm, and 10 in the 45 mg binimetinib intermittent arm were assessed for response (Table 3). Three patients, all on the 45 mg binimetinib continuous arm, were not assessed for response due to the following reason: suicide on cycle 1 (1), death secondary to acute cardiopulmonary arrest on cycle 4 prior to first re-staging scan (1), hypersensitivity to oxaliplatin of cycle 1 (1). Nine out of 13 assessed patients on the continuous binimetinib dosing experienced stable disease (≥ 2 months), with one patient maintaining SD for 9 months. In contrast, 9 out 10 patients on the 45 mg binimetinib intermittent dosing experienced disease progression (PD) at 2 months, with the remaining patient progressing on the second evaluation at 4 months (Figure 1).

**Table 3:** Efficacy

Dosing schedule	Continuous arm 30 mg binimetinib BID + FOLFOX *n* = 3	Continuous arm 45 mg binimetinib BID + FOLFOX *n* = 13	Intermittent arm 45 mg binimetinib BID + FOLFOX *n* = 10
Median duration of treatment (cycles)	8 (range 3–12)	5 (range 1–20)	4 (range 4–8)
Number of cycles	6 (range 1–20)		4 (range 4–8)
Best overall response CR PR SD** PD NA	0 0 2 1 0	0 0 7^#^ 3 3*	0 0 1 9
Clinical benefit rate (CR + PR + SD)	66.6%	54%	10.0%
Median PFS (months)	5.5 (95% CI 1.41- NR)	3.5 (95% CI 1.9-NR)	1.8 (95% CI 1.7-NR)
Median PFS (months)	3.5 months (95% CI 1.9-NR)		1.8 (95% CI 1.7-NR)

BID, twice daily; FOLFOX, 5-flourouracil, leucovorin, and oxaliplatin; CR, complete response; PR, partial response; SD, stable disease; PD, disease progression; PFS, progression-free survival; CI, confidence interval. *1 suicide, 1 early death considered a cardiac event, 1 off early for toxicity. **SD for at least 1 evaluation. ^#^Prolonged SD of 9 months in 1 patient. 4 patients were censored for PFS at time of treatment discontinuation due to toxicity (2), surgery (1), and patient choice (1).

The median time to treatment failure at the MTD of the continuous and intermittent binimetinib arms were 3.5 months (95% CI 1.9-NR) and 1.8 months (95% CI 1.7-NR), respectively. The longest time to treatment failure of 272 days was observed in a patient with wild-type *KRAS*, MSI high mCRC, previously treated with 3 lines of prior therapy, never was on immunotherapy previously, and enrolled on the 45 mg binimetinib continuous dosing arm (Figure 1).

### Pharmacokinetics

Six patients were enrolled and treated at the defined MTD of 45 mg oral twice daily binimetinib continuous dosing + FOLFOX every 2 weeks to investigate potential PK interactions between 5-FU, oxaliplatin, and binimetinib (Table 4). The average steady-state concentration of 5-FU without binimetinib was 510.6 ±120.2 ng/mL and was comparable to that of 5-FU given with binimetinib (597.6 ± 204.5 ng/mL). The average Cmax, AUC, and T_1/2_ of oxaliplatin when given without binimetinib were 598.8 ± 148.9 μg/L, 26580.5 ± 4844.8 μg/L × hr, and 37.1 ± 11.7 hr compared to 663.9 ± 75.9 μg/L, 32190.9 ± 11861.2 μg/L × hr, and 38.8 ± 22.6 hr when given with binimetinib. In this expanded MTD cohort, no patients experienced DLTs. PK analyses were not conducted for the intermittent binimetinib dosing schedule.

**Table 4:** Pharmacokinetics

		Steady-state level (ng/mL) Cycle 1 (without binimetinib)	Steady-state level (ng/mL) Cycle 2 (with binimetinib)
5-FU	Pt#1 Pt#2 Pt#3 Pt#4 Pt#5 Pt#6	353.0 524.0 627.5 427.5 621.0 428.0	293.1 480.5 718.0 770.5 726.0 ND
	Avg STD	510.6 120.2	597.6 204.5

		Cmax (μg/L)	AUC (μg/L X hr)	T_1/2_ (36)	Cmax (μg/L)	AUC (μg/L X hr)	T_1/2_ (36)
	Cycle 1 (without binimetinib)			Cycle 2 (with binimetinib)			
Oxaliplatin	Pt#1 Pt#2 Pt#3 Pt#4 Pt#5 Pt#6	536.0 780.8 686.5 601.5 389.3 ND	28618.0 31146.9 25757.7 18615.5 28764.2 ND	56.7 32.7 30.6 27.0 38.3 ND	ND 590.0 726.5 748.5 583.5 671.0	ND 51036.0 33953.4 25138.7 19805.3 31020.9	ND 77.9 38.9 26.4 26.3 24.7
	Avg STD	598.8 148.9	26580.5 4844.8	37.1 11.7	663.9 75.9	32190.9 11861.2	38.8 22.6

5-FU, 5-flourouracil; ND, not determined; STD, standard deviation; Cmax, maximum serum concentration; AUC, area under the curve; T_1/2_, half-life.

## DISCUSSION

The objective of this first in-human, open-label, single-arm, dose-escalation phase I study was to determine the safety and feasibility of combining oral binimetinib with fixed-dose FOLFOX every 2 weeks in individuals with metastatic or advanced CRC who have progressed on prior standard therapies. Out of 26 patients enrolled in this study, the combination of binimetinib (continuous and intermittent dosing schedules) and FOLFOX was generally well tolerated. Following standard dose-escalation rules, no DLTs were encountered in all binimetinib dosing arms and the MTD of binimetinib in combination with FOLFOX every 2 weeks was 45 mg orally twice daily in either continuous or intermittent dosing schedules. Common DLTs that have been observed in early binimetinib trials include CPK elevation, retinopathy, and skin toxicity, which appear to represent class effects of MEK inhibitors [15–17, 19–22]. Through baseline and every 2-cycle ophthalmologic examinations, we encountered only 1 case of a non-DLT of grade 3 retinopathy observed in a 59-year-old Caucasian male following 8 cycles of study treatment. He had no prior risk factors and experienced resolution of his RVO nearly 3 months later following discontinuation of binimetinib 30 mg twice daily continuous dosing. Three events of asymptomatic grade 3 CPK elevation occurred on the continuous daily dosing of binimetinib. CPK elevations resolved after discontinuation of binimetinib. Of interest, two patients were noted to have radiographic findings concerning for nephritis on imaging studies. One patient had an associated grade 1–2 CPK elevation, while the second had an associated grade 3 CPK elevation. Neither case of radiographic renal changes was associated with renal dysfunction or urinalysis abnormalities. To the best of our knowledge, there is sparse evidence of MEK inhibitor-associated renal damage in the literature. One case of interstitial nephritis has been described in a patient with locally advanced melanoma treated with the MEK 1/2 inhibitor trametinib in combination with dabrafenib [26]. For binimetinib therapy, the occurrence of cardiac events are relatively rare and were seen in 1 patient with atrial fibrillation (out of 22 with advanced *NRAS* melanoma) and 1 patient with irregular heart rate (out of 30 with advanced *NRAS* melanoma) across 2 separate early-stage studies (18, 22). We experienced two cases with cardiac adverse events on our study. One case consisted of elevated troponin levels in association with an oxaliplatin-induced anaphylactic reaction. The other case consisted of cardiac arrest and hypoxia on cycle 4 of treatment. Direct causality to study treatment could not be confirmed in that case. No clinically significant decrease in ejection fraction was noted on study.

PK analysis of 5-FU and oxaliplatin with and without binimetinib was performed at the MTD of 45 mg twice daily binimetinib continuous dosing. There were no significant differences in either 5-FU or oxaliplatin PK parameters when these agents were given with and without oral binimetinib administration. Moreover, 5-FU and oxaliplatin PK data were comparable to previously published results with FOLFOX alone [27]. Our findings are consistent with PK analyses from other phase I studies that similarly reported no evidence of drug-drug interactions when binimetinib is combined with conventional chemotherapy [19, 25].

We proposed that MEK inhibition may improve clinical responses and overcome resistance to platinum-based (FOLFOX) therapy in mCRC based on preclinical evidence [7–10, 28–31]. In our cohort of metastatic or advanced CRC patients who progressed following fluoropyrimidine, irinotecan, and oxaliplatin-based chemotherapy, treatment with continuous binimetinib + FOLFOX every 2 weeks produced a promising SD rate of 69% in 13 evaluable patients. Furthermore, the median PFS was 3.5 months (95% CI 1.9- NR) in the MTD cohort of continuous binimetinib + FOLFOX. In phase I trials, single-agent binimetinib produced SD rates as high as 67% in patients with advanced solid tumors [15–17]. The activity noted in RAS and BRAF wild-type patients in our study could be related to possibly enrolling MEK sensitive RAS-WT and BRAF-WT tumors. In addition, all our RAS-WT and BRAF-WT colorectal cancer patients were anti-EGFR resistant, a setting associated with MAPK pathway activation [32]. It is therefore possible that these anti-EGFR resistant RAS and BRAF-WT tumors are particularly sensitive to MEK inhibition. The promising activity with continuous binimetinib 45 mg twice daily + FOLFOX every 2 weeks in an otherwise heavily pretreated population (69.2% having received ≥ 3 prior lines of therapies) warrants further investigation in larger prospective trials in mCRC patients. Currently, the BEACON phase III clinical trial (NCT02928224) is also evaluating the role of binimetinib in combination with a BRAF and EGFR inhibitor in BRAF mutated colorectal cancer patients.

Notably, the longest PFS of 272 days was observed in a patient with wild-type *KRAS*, MSI high mCRC who progressed following three prior lines of treatment and who had not been on immunotherapies prior to enroll in this study. Sustained response has also been identified the addition of the MEK 1/2 inhibitor trametinib in a patient with heavily pretreated MSI high (MSI-H) metastatic endometrial cancer [33]. There is growing interest in the potential immunogenicity of MEK inhibitors in mCRC. A recent phase Ib study investigated escalating doses of the MEK inhibitor cobimetinib in combination with the checkpoint inhibitor atezolizumab in 23 patients with previously-treated mCRC [34]. Partial responses were seen in 4 patients (17%), including 3 patients having microsatellite stable (MSS) tumors. Whether MEK inhibition enhances immune responses to checkpoint inhibitors differently between MSS and MSI-H mCRC remains to be determined, though future investigations are likely to further expand on this novel concept.

The rationale for investigating an intermittent binimetinib dosing schedule arose from preclinical data supporting that pretreatment with a MEK inhibitor resulted in synergistic antitumor activity in cancer cell lines as well as *in vivo* data showing that intermittent binimetinib dosing enhanced the activity of conventional cytotoxic agents [7, 14]. In contrast, to the continuous binimetinib cohort, minimal clinical activity was noted with intermittent binimetinib dosing, with 9/10 patients progressing on their first 2-month imaging scans. These findings support the need of sustained MAPK inhibition with continuous binimetinib dosing to result in optimal tumor inhibition. Indeed, clinical activity with binimetinib monotherapy in other tumor types have only been reproduced with continuous dosing [15–18].

In conclusion, the combination of continuous oral binimetinib and FOLFOX every 2 weeks is safe and feasible in the treatment of patients with metastatic or advanced CRC who have progressed on prior standard therapies. There were no clinically significant differences in PKs and no evidence of drug-drug interactions when binimetinib is combined with 5-FU and oxaliplatin. Continuous binimetinib at the MTD of 45 mg oral twice daily with FOLFOX every 2 weeks showed promising activity in a heavily pretreated population of mCRC patients. Further evaluation is warranted in larger prospective trials involving mCRC patients, particularly in patients where anti-EGFR therapy is contraindicated (RAS and BRAF mutations).

## MATERIALS AND METHODS

### Study population

This was a first in-human, single-arm, open-label, dose-escalation phase I study investigating the safety and tolerability of two dosing schedules of oral binimetinib (continuous and intermittent dosing) in combination with fixed-dose FOLFOX in patients with metastatic or advanced CRC who progressed on prior standard cytotoxic treatments and anti-EGFR therapy. The study was conducted from July 2014 to February 2016 at the City of Hope Comprehensive Cancer Center (COH; Duarte, CA). All patients were adults ≥ 18 years with pathologically confirmed colon or rectal cancer who progressed following fluoropyrimidine, irinotecan, and oxaliplatin-based chemotherapy. Patients with known wild-type *KRAS*/*BRAF* tumors should have progressed following cetuximab or panitumumab-based therapy. Prior regorafenib or bevacizumab exposure was not mandated. Eligible patients must have also had the following: measurable disease defined as a minimum of one tumor ≥ 10 mm on computed tomography (CT) scan, absolute neutrophil count (ANC) ≥ 1.5 × 10^9^/L, hemoglobin (Hgb) ≥ 9 g/dL without transfusion, platelets (PLT) ≥ 100 × 10^9^/L without transfusion, *aspartate transaminase* (AST) and/or *alanine transaminase* (ALT] ≤ 2.5 × upper limit of normal (ULN) in the absence of liver metastases and ≤ 5 × ULN in the presence of liver metastases, total bilirubin ≤ ULN, creatinine ≤ 1.5 mg/dL, left ventricular ejection fraction (LVEF) ≥ 50% via multigated acquisition (MUGA) scan or echocardiogram, QTc interval ≤ 480 ms, ability to sign informed consent and take oral medications, means to be compliant with protocol, negative serum beta-human chorionic gonadotropin (β-hCG) within 72 hours prior to first dose, and Eastern Cooperative Oncology Group (ECOG) performance status (PS) ≤ 1.

Patients were deemed ineligible if they had the following: history or current evidence of retinal vein occlusion (RVO) or current risk factors for RVO (e.g., uncontrolled glaucoma or ocular hypertension, history of hyperviscosity, or hypercoagulability syndromes), prior chemotherapy, biologic, targeted, or radiotherapy within 4 weeks prior to entering study or not recovered from grade ≥ 2 AEs due to agents administered more than 4 weeks earlier (except alopecia or neuropathy), history of retinal degenerative disease, history of Gilbert’s syndrome, previous or concurrent malignancy (except for adequately treated and healed basal cell or squamous cell carcinoma of the skin, *in situ* carcinoma of the cervix treated curatively and without evidence of recurrence, or primary malignancy completely resected and in complete remission ≥ 1 year), prior MEK inhibitor therapy, history of acute coronary syndromes < 6 months prior to screening, impaired cardiovascular function or clinically significant cardiovascular disease, uncontrolled arterial hypertension despite appropriate medical therapy (systolic blood pressure > 160 or diastolic blood pressure > 100), known positive serology for HIV, active hepatitis B, and/or active hepatitis C, neuromuscular disorders associated with elevated creatine phosphokinase, started or planning to start on strenuous exercise regimen after first dose of study treatment, gastrointestinal (GI) disease or impairment of GI function (e.g., active ulcerative disease, uncontrolled nausea, vomiting, or diarrhea, malabsorption syndrome, or small bowel resection), prior major surgery ≤ 3 weeks before study drug or not recovered from side effects of such procedure, pregnant or lactating women, use of other investigational drugs, grade ≥ 3 neuropathy, known hypersensitivity to any components of study drugs, prior intolerance to 5-FU or oxaliplatin (except neuropathy that reversed to grade ≤ 2), and any other condition (medical, psychiatric, or cognitive) deemed by investigator to contraindicate patient’s participation in study due to safety concerns, compliance, ability to give informed consent, or ability to complete study.

The study was conducted in full compliance as outlined by the COH Institutional Review Board. The study was carried in adherence to the principles of Good Clinical Practice as outlined in Title 21 of the U.S. Code of Federal Regulations and the Declaration of Helsinki. Investigators obtained informed consent from each participant and this study was registered as NCT02041481.

### Study design

A standard 3 + 3 dose-escalation design was followed using a continuous and intermittent oral binimetinib dosing schedule combined with fixed-dose FOLFOX. A fixed-dose FOLFOX regimen on both schedules consisted of LV 400 mg/m^2^ 2-hour infusion concurrently with oxaliplatin 85 mg/m^2^ infusion followed by 5-FU 2400 mg/m^2^ infusion over 46 hours (no bolus 5-FU ) every 2 weeks. The continuous binimetinib dosing schedule was comprised of 2 dosing levels: 30 mg orally twice daily and 45 mg orally twice daily continuously starting on day 1 with FOLFOX, repeated every 2 weeks. The MTD of binimetinib continuous daily dosing was explored in an additional 6-patient cohort to investigate potential FOLFOX-binimetinib pharmacokinetic interactions. In addition, the MTD dose of binimetinib was explored further with FOLFOX in an intermittent sequential schedule in order to minimize treatment toxicity, with binimetinib twice daily on days 1–5 with FOLFOX on days 6–7 every 2 weeks. The starting dose of binimetinib on the intermittent arm was the MTD dose level identified on the continuous dosing schedule. Following standard dose-escalation rules, the maximum tolerated dose (MTD) for either schedule was defined as the highest dose level tested in which no more than 1 of 6 patients experienced a dose-limiting toxicity (DLT) in the dose-escalation phase. At least 6 patients must have been treated at the MTD and the evaluation of DLTs and MTD was based on first 2 cycles of study treatment (4 weeks). No intra-patient dosing escalations were allowed. No escalation beyond the previous binimetinib monotherapy recommended dose of 45 mg PO BID was allowed. The MTD cohort on the continuous binimetinib dosing included 12 patients evaluable for DLT consideration (6 patients in the escalation phase and 6 in the PK cohort). The MTD cohort on the intermittent arm was expanded to 10 patients in order to have a more robust assessment of the toxicity of this schedule.

DLTs were defined as any toxicity (graded according to Common Terminology Criteria for Adverse Events or CTCAE version 4.03) occurring within the first 2 cycles of treatment deemed as at least possibly related to treatment and meeting the following criteria: any grade ≥ 3 non-hematologic toxicity (grade 3 exceptions: oxaliplatin-induced neuropathy that resolves within 2 weeks; oxaliplatin-related hypersensitivity; rash < 1 week; diarrhea, nausea, or vomiting < 48 hours; fatigue or edema < 5 days; laboratory abnormalities that correct to grade ≤ 2 within 24 hours), grade 2 retinopathy lasting 14 days confirmed on ophthalmologic exam or any grade ≥ 3 retinopathy, QTc interval ≥ 501 ms on at least 2 separate electrocardiograms from the same visit, serum creatinine > 2× ULN that does not reverse with hydration, grade 4 thrombocytopenia, grade 4 neutropenia > 7 days, or grade ≥ 3 neutropenia with fever (temperature ≥ 38.5 degrees Celsius). No dose adjustments were allowed in the first 2 cycles of treatment, with the exception for DLTs which required treatment interruption. Starting cycle 3 and beyond, patients who did not tolerate 45 mg oral twice daily could be de-escalated to 30 mg twice daily; reduction below 30 mg twice daily required study discontinuation.

Patients’ response to treatment were assessed by radiographic imaging every 8 weeks in accordance to the revised Response Evaluation Criteria in Solid Tumors (RECIST) guideline, version 1.1 [35]. An ophthalmological exam was done at screening, during cycle 2 of treatment, and every 2 cycles from there on. An echocardiogram or MUGA scan was performed at 4 weeks and 8 weeks after study initiation and every 12 weeks thereafter. Study treatment was continued until disease progression, intercurrent illness preventing further therapy, unacceptable AEs, patient withdrawal, or changes in the patient’s condition that renders the patient at risk from further therapy. Patients were followed for toxicity up to 30 days of study treatment discontinuation.

### Endpoints

The primary endpoint of this study was to evaluate the MTD for the combination of binimetinib and FOLFOX in mCRC patients. Secondary objectives included an assessment of safety and toxicity of this combination across all investigated dosing levels, pharmacokinetics (PKs) of continuous binimetinib dosing and FOLFOX in an expanded MTD cohort, and any evidence of clinical activity with this combination per RECIST guideline 1.1.

### Pharmacokinetics

Six patients were enrolled at the continuous binimetinib MTD dose-level to investigate potential PK interactions between 5-FU, oxaliplatin, and binimetinib. In this expanded cohort, continuous binimetinib did not start until day 4 of cycle 1 to obtain PKs of 5-FU and oxaliplatin without binimetinib. Blood samples for 5-FU and oxaliplatin determination was performed on days 1–2 of cycles 1 (without binimetinib) and 2 (with binimetinib).

### Fluorouracil and oxaliplatin

Blood was collected for PK analysis at the initiation of the oxaliplatin infusion (time 00:00) and at the following time points 01:00, 01:30, 02:00, 04:00, 08:00, 24:00, and 44:00-46:00 (prior to end of 5-FU infusion). Oxaliplatin levels in plasma and ultrafiltrate were determined by atomic absorption spectrophotometry at the City of Hope Analytical Pharmacology Core Facility (APCF) with lower limit of quantitation (LLOQ) in ultrafiltrate and plasma of 10 ng/mL and a lower limit of detection (LLOD) of 5 ng/mL. Fluorouracil levels in plasma were determined using an LC-MS/MS analytical assay also in the APCF with LLOQ of 3 ng/mL from a 200 μL aliquot of plasma.

### Statistical analysis

No formal statistical hypotheses were assessed. Patient demographics including age, sex, race, location of primary tumor, ECOG PS, *RAS* mutation status, and PK parameters were reported using descriptive statistics as counts, means, medians, and percentages. Toxicities were tabulated according to dose, type, and grade. Clinical responses were reported as per RECIST guideline 1.1. Progression-free survival (PFS) was time from start of treatment to either death or progression (otherwise patients were censored at stop of protocol treatment). Time to treatment failure was from start of treatment to end of treatment (no censoring). Kaplan-Meier methods were used to evaluate PFS. Calculations were carried out using S-Plus 8.2.
